# Multivariate analysis for identifying drought-tolerant barley (*Hordeum vulgare* L.) genotypes using stress indices

**DOI:** 10.1016/j.dib.2025.111452

**Published:** 2025-03-07

**Authors:** Nadira Mokarroma, Md Romij Uddin, Imrul Mosaddek Ahmed, AHM Motiur Rahman Talukder, Abul Fazal Mohammad Shamim Ahsan, Zakaria Alam

**Affiliations:** a Plant Physiology Division, Bangladesh Agricultural Research Institute, Gazipur, 1701, Bangladesh; b Department of Agronomy, Bangladesh Agricultural University, Mymensingh 2202, Bangladesh; c Tuber Crops Research Centre, Bangladesh Agricultural Research Institute, Gazipur, 1701, Bangladesh

**Keywords:** Drought, Factor analysis, Stress indices, MGIDI, Broad sense heritability

## Abstract

The stress indicator widely expresses the impression of drought stress on barley genotypes throughout the crown root initiation period, highlighting its worldwide effect on production. So, it was urgent need to identify the drought-resilient genotypes considering the multi-trait genotype ideotype distance index (MGIDI). The study, conducted to evaluate the grain output and genetic variations of 50 barley genotypes using stress indicator. Under optimal conditions, genotype IBON14 achieved the highest grain yield of 9.55 g/plant, while under drought stress, genotype BD7194 produced 7.31 g/plant. Significant correlations, both positive and negative (ranging from 0.66 to 1.00), were observed among stress tolerance indices and yields. Using the MGIDI index, genotype BD7194 was selected as the most drought-tolerant, followed by BD7188, IBON14, BD8579, and IBON16, with a 5 % selection intensity. Factor study among the MGIDI revealed diverse tolerance and susceptibility indices, emphasizing the robustness of the certain genotypes, all of which were grouped under a single factor. The selected genotypes exhibited a selection gain (%) between 39.9 % and 113 %. Moreover, the selection differential, calculated from predicted values, varied from 0.25 to 2.54, and broad-sense heritability was determined to be ≥0.99. This study emphasizes the usefulness of the MGIDI index in selecting drought-tolerant barley genotypes, with BD7194 proving to be the most resilient, exhibiting high genetic stability and selection gains.

Specifications Table

**Table:** 

Subject	Agricultural and Biological Science
Specific subject area	Agronomy and Crop Science
Data format	Raw
Type of data	Table and Figures
How the data were collected	The grain yield data for both stressed and non-stressed plants were measured using an electrical balance. The whole grain weight (in grams) was recorded for each genotype across all replications, and the average values were used for subsequent analysis.
Data source location	Barley genotypes were examined for drought tolerance in the vinyl house of the Plant Physiology Division at the Bangladesh Agricultural Research Institute (BARI) in Gazipur, Bangladesh.
Data accessibility	Repository name: Mendeley Data Direct URL to data: https://data.mendeley.com/datasets/gmrzt4f99w/1

## 1 Value of the Data

• The dataset offers valuable insights into the impact of drought stress on barley yield, particularly during the crown root initiation stage. Understanding this stress's effect on crop productivity is crucial for developing effective crop management strategies that benefit both the global barley industry and producers.
• Analysed through the MGIDI index, the dataset reveals worthy information on genotypes exhibiting drought resilience. The findings underscore the importance of using multivariate analysis to assess yields and identify drought-resistant barley genotypes, which is vital for breeding programs aimed at improving barley yields in drought-prone areas.
• The dataset emphasizes critical factors in identifying drought-tolerant barley genotypes from a diverse assortment of germplasm. The selected genotypes offer practical benefits through the use of metrics such as broad-sense heritability, selection differential, and selection gain, which deliver a quantifiable assessment of data reliability and highlight their potential influence on future breeding efforts.

## 2 Background

Environmental stresses such as heat and drought are significant factors that often restrict the growth and yield of many crops [1]. Numerous studies have highlighted the genetic variability in drought sensitivity both within and across crop species [2]. Barley (*Hordeum vulgare* L.), ranked as the fourth most important cereal crop following wheat, corn, and rice, is cultivated to provide nutrition for livestock, humans, and the brewing sector. As an ancient and versatile grain, barley has played a vital role in human history, significantly contributing to agriculture, nutrition, and culture [3]. However, drought stress has been shown to reduce barley grain yield by 49–87 % [4]. Therefore, breeding drought-tolerant barley varieties is the most practical and economical method to reduce the detrimental effects of drought on barley yield. With climate change potentially intensifying these stresses, breeders face increased challenges in selecting suitable genotypes. The concept of a plant ideotype [[5], [6], [7]], which takes into account various traits [[8], [9], [10], [11], [12], [13], [14]], can aid in selecting stress-tolerant genotypes within a given collection.

### 2.1 Data explanation

This dataset contains two tables and two figures. Table 1 presents the drought tolerance and susceptibility indices, as well as the grain yield for 50 barley genotypes. Under normal conditions, genotype IBON 14 achieved the highest grain yield of 9.55 g plant⁻¹, followed by BD7194 with 8.95 g plant⁻¹ and BD7188 with 8.53 g plant⁻¹. In drought stress conditions, BD7194 produced the highest yield at 7.31 g plant⁻¹, while IBON 14 and BD7188 yielded 7.02 g plant⁻¹ and 6.90 g plant⁻¹, respectively.

**Table 1:** Stress indices of fifty barley genotypes under control and stress condition along with the yield.

Genotypes	Yp (g plant^-1^)	Ys (g plant^-1^)	TOL	MP	GMP	SSI	STI	YSI	YI
BD7194	8.95	7.31	1.65	8.13	8.09	0.40	1.37	0.82	1.95
IBON14	9.55	7.02	2.53	8.29	8.19	0.58	1.41	0.74	1.88
BD7188	8.53	6.90	1.62	7.71	7.67	0.42	1.23	0.81	1.85
BD8579	7.72	6.09	1.63	6.91	6.86	0.46	0.99	0.79	1.63
IBON16	8.10	6.04	2.06	7.07	6.99	0.55	1.02	0.75	1.61
BD9681	8.43	6.00	2.43	7.22	7.11	0.63	1.06	0.71	1.60
IBON59	7.46	5.60	1.86	6.53	6.46	0.54	0.88	0.75	1.50
IBON120	7.92	5.57	2.35	6.75	6.65	0.65	0.93	0.70	1.49
BD7205	7.56	5.45	2.11	6.50	6.42	0.61	0.86	0.72	1.46
BD7192	7.50	5.38	2.12	6.44	6.35	0.62	0.84	0.72	1.44
IBON13	7.35	5.21	2.14	6.28	6.19	0.64	0.80	0.71	1.39
BD7196	7.09	5.13	1.95	6.11	6.03	0.60	0.76	0.72	1.37
BD7189	6.87	4.94	1.93	5.90	5.82	0.61	0.71	0.72	1.32
BD7191	6.79	4.86	1.93	5.83	5.75	0.62	0.69	0.72	1.30
IBON28	7.92	4.86	3.06	6.39	6.20	0.84	0.81	0.61	1.30
BARIBarley9	7.00	4.71	2.29	5.86	5.74	0.71	0.69	0.67	1.26
BARIBarley8	6.56	4.57	1.99	5.57	5.48	0.66	0.63	0.70	1.22
IBON19	7.20	4.25	2.94	5.72	5.53	0.89	0.64	0.59	1.14
BD9680	7.07	4.16	2.91	5.61	5.42	0.90	0.62	0.59	1.11
IBON37	7.04	3.92	3.11	5.48	5.25	0.96	0.58	0.56	1.05
BD7203	6.99	3.87	3.12	5.43	5.20	0.97	0.57	0.55	1.04
BD7202	6.99	3.82	3.18	5.41	5.17	0.99	0.56	0.55	1.02
IBON54	6.97	3.63	3.35	5.30	5.03	1.05	0.53	0.52	0.97
BD9194	6.97	3.45	3.51	5.21	4.91	1.10	0.50	0.49	0.92
IBON36	6.90	3.34	3.56	5.12	4.80	1.12	0.48	0.48	0.89
BD8574	6.97	3.34	3.63	5.16	4.83	1.13	0.49	0.48	0.89
BD7193	6.85	3.19	3.66	5.02	4.68	1.16	0.46	0.47	0.85
IBON8	6.75	3.08	3.67	4.92	4.56	1.19	0.44	0.46	0.82
IBON52	6.68	2.95	3.73	4.82	4.44	1.22	0.41	0.44	0.79
BD8572	6.48	2.80	3.68	4.64	4.26	1.24	0.38	0.43	0.75
IBON24	6.59	2.80	3.80	4.70	4.29	1.26	0.39	0.42	0.75
BD7204	6.56	2.77	3.79	4.67	4.27	1.26	0.38	0.42	0.74
BD7206	6.50	2.70	3.80	4.60	4.19	1.28	0.37	0.42	0.72
BARIBarley2	6.47	2.63	3.83	4.55	4.13	1.29	0.36	0.41	0.70
IBON68	6.38	2.59	3.79	4.48	4.06	1.29	0.35	0.41	0.69
BD9682	6.17	2.54	3.63	4.36	3.96	1.28	0.33	0.41	0.68
IBON11	6.52	2.45	4.07	4.49	4.00	1.36	0.33	0.38	0.66
BD9683	6.51	2.33	4.18	4.42	3.89	1.40	0.32	0.36	0.62
BD7088	6.35	2.29	4.06	4.32	3.81	1.39	0.30	0.36	0.61
IBON21	6.16	2.29	3.87	4.22	3.76	1.37	0.30	0.37	0.61
IBON26	6.40	2.29	4.11	4.34	3.83	1.40	0.31	0.36	0.61
IBON86	6.30	2.25	4.05	4.28	3.77	1.40	0.30	0.36	0.60
IBON12	6.12	2.20	3.92	4.16	3.67	1.39	0.28	0.36	0.59
BARIBarley5	5.99	2.16	3.83	4.07	3.60	1.39	0.27	0.36	0.58
BD8573	6.27	2.15	4.11	4.21	3.67	1.43	0.28	0.34	0.58
BD7195	5.87	1.95	3.92	3.91	3.38	1.46	0.24	0.33	0.52
BD9684	6.34	1.80	4.53	4.07	3.38	1.56	0.24	0.28	0.48
IBON9	5.21	1.80	3.41	3.50	3.06	1.43	0.20	0.35	0.48
BD7197	5.30	1.76	3.53	3.53	3.06	1.45	0.20	0.33	0.47
IBON97	6.45	1.63	4.82	4.04	3.24	1.63	0.22	0.25	0.44

^Yp^ Yield under control condition, ^Ys^ yield under stress condition, ^TOL^ Tolerance Index, ^MP^ Mean Productivity, ^GMP^, Geometric Mean Productivity mean, ^SSI^ Stress Susceptibility Index, ^STI^ Stress Tolerance Index, ^YSI^ Yield stability index and ^YI^ Yield Index.

Table 2 presents a factor analysis that examines the drought tolerance and susceptibility indices for the various genotypes. Factor 1 (FA1) encompasses all indices, each demonstrating a 100 % success rate, which indicates no weaknesses among the genotypes. These genotypes exhibit a selection gain ranging from 39.9 % to 113 %, while the selection differential fluctuates between 0.25 and 2.54. Broad-sense heritability was notably high, at or above 0.99.

**Table 2:** Factor contribution (FA), observed mean ($\bar{X}$_o_), predicted mean ($\bar{X}$_s_), broad-sense heritability (h^2^), selection differential (SD) and selection gain (SG) obtained using MGIDI of fifty barley genotypes.

Parameter	Factor	$\bar{X}$_o_	$\bar{X}$_s_	SD	h^2^	SG (%)	Sense	Goal
TOL	FA1	3.18	1.9	-1.27	0.99	-39.9	decrease	100
MP		5.33	7.62	2.3	0.99	43.1	increase	100
GMP		5.02	7.56	2.54	0.99	50.5	increase	100
SSI		1.04	0.48	-0.55	0.99	-53.3	decrease	100
STI		0.57	1.2	0.64	1	113	increase	100
YSI		0.53	0.78	0.25	0.99	48	increase	100
YI		0.99	1.78	0.79	0.99	78.5	increase	100

^TOL^ Tolerance Index, ^MP^ Mean Productivity, ^GMP^ Geometric Mean Productivity mean, SSI Stress Susceptibility Index, STI Stress Tolerance Index, YSI Yield stability index and ^YI^ Yield Index.

**Fig. 1:**
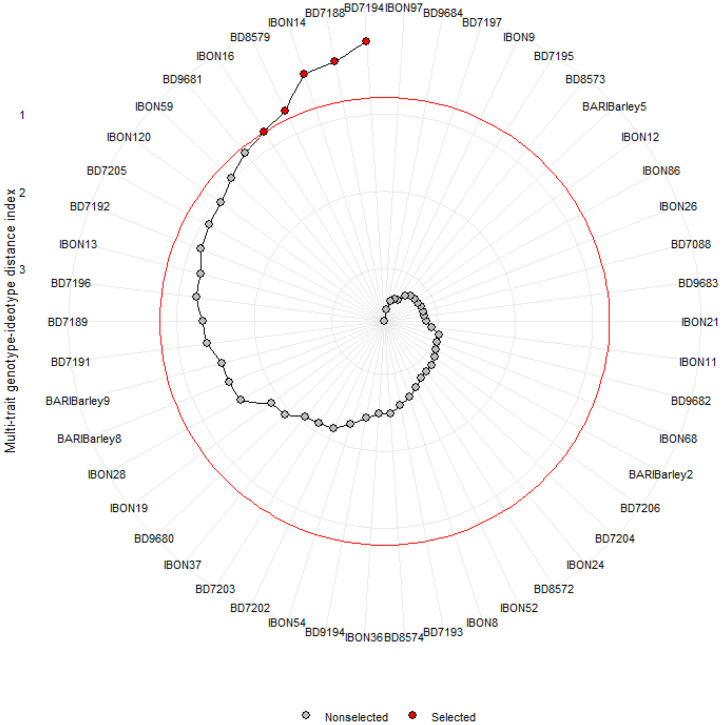
Selection of drought tolerant barley genotypes through multi trait genotype ideotype distance index (MGIDI).

Fig. 1 displays the MGIDI index structured based on the traits of seven stress indices for 50 barley genotypes. Among these genotypes, BD7194 is identified as the top-ranked, followed by BD7188, IBON 14, BD8579, and IBON 16.

**Fig. 2:**
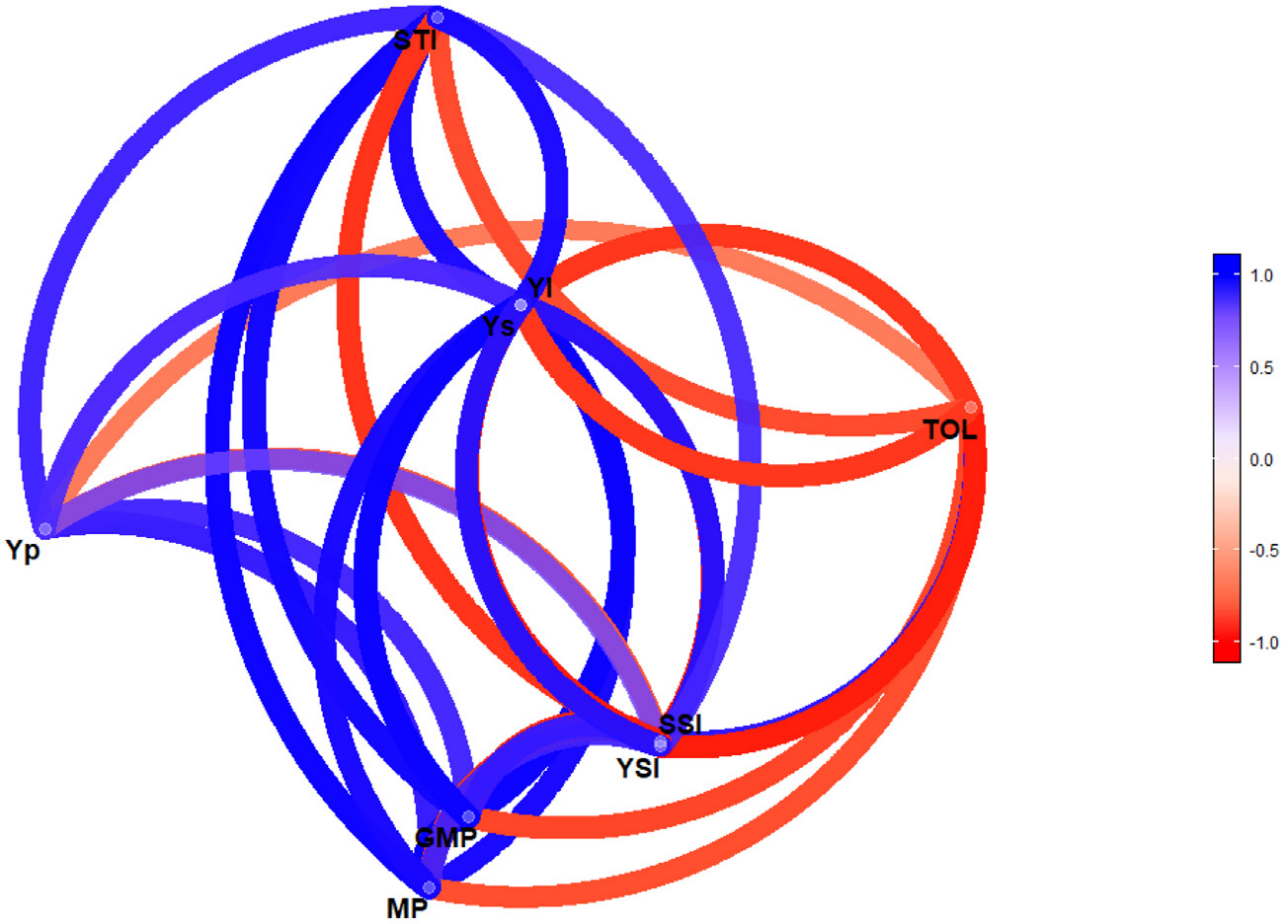
Network plot of Pearson's correlation coefficients between Y_P_ (Control), Y_S_ (Drought) and drought tolerance indices. ^Yp^ Yield under control condition, ^Ys^ yield under stress condition, ^TOL^ Tolerance Index, ^MP^ Mean Productivity, ^GMP^ Geometric Mean Productivity mean, ^SSI^ Stress Susceptibility Index, ^STI^ Stress Tolerance Index, ^YSI^ Yield stability index and ^YI^ Yield Index

Fig. 2 illustrates a network plot showing the Pearson's correlation coefficients between yields and the drought tolerance indices. The network plot demonstrates a significant correlation (p<0.001; r= -0.66 to 1.00) between the traits analysed, with the correlation coefficients and significance levels detailed in Table S1. A positive and significant correlation was observed between TOL and SSI, Yp and TSI, Yp and Ys, Yp and YI, Yp and STI, Yp and MP, Yp and GMP, YSI and Ys, YSI and YI, YSI and STI, YSI and MP, YSI and GMP, Ys and YI, Ys and STI, Ys and MP, Ys and GMP, YI and STI, YI and MP, YI and GMP, STI and MP, STI and GMP, and MP and GMP. Conversely, significant negative correlations were found between SSI and Yp, SSI and YSI, SSI and Ys, SSI and YI, SSI and STI, SSI and MP, SSI and GMP, TOL and Yp, TOL and YSI, TOL and Ys, TOL and YI, TOL and STI, TOL and MP, and TOL and GMP (Fig. 2).

## 3 Experimental Design, Materials and Methods

### 3.1 Study treatments, plan and genetic materials

In this experiment, drought treatment was implemented during the vegetative stage and consisted of two conditions: (1) a control (no drought), in which soil moisture was maintained at 90–100 % of field capacity, and (2) a drought condition, where irrigation was stopped from crown root initiation (CRI) until just before anthesis, resulting in soil moisture dropping to 40–45 % of field capacity. Soil moisture was monitored every other day using a moisture meter (HH2, Delta-T Devices, Cambridge, England). The study included 50 barley genotypes, with ten seeds sown in each pot, and each pot was replicated three times. Thinning was performed 10 days after sowing, leaving five plants per pot until harvest. The experiment was conducted using a randomized complete block design (RCBD). The details of studied barley genotypes are placed in Table S2.

### 3.2 Experimental location and techniques

A drought tolerance screening of barley genotypes was conducted in the vinyl house of the Plant Physiology Division at the Bangladesh Agricultural Research Institute (BARI), Gazipur, from November 2018 to March 2019. Agricultural soil was collected from the central experimental farm at BARI (depth 0–15 cm), sun-dried, and mixed daily until it reached a moisture content of 8 %, measured by a moisture meter. Plastic pots with a 6 kg capacity (20 cm top diameter, 15 cm base diameter, and 19 cm height) were filled with a mixture of sun-dried sandy loam soil and cow dung in a 4:1 ratio. The soil was clay loam with an acidic pH of 6.1. Fertilizer was applied to the pots at rates of 120 kg ha⁻¹ triple super phosphate, 110 kg ha⁻¹ muriate of potash, 50 kg ha⁻¹ gypsum, and two-thirds of 160 kg ha⁻¹ urea three days before sowing. The remaining third of the urea was applied 20 days after sowing at the crown root initiation (CRI) stage.

### 3.3 Data assortment and analysis

At the harvesting, three replicates of plants were collected for biomass measurement, and grain yield data for 50 barley genotypes were recorded. The grain yield (in grams) for each genotype (five plants) was measured under both control (Yp) and stress (Ys) conditions. These measurements were subsequently averaged. Seven indices were calculated to evaluate stress tolerance and susceptibility, including Tolerance Index (TOL) [15], Mean Productivity (MP) [15], Geometric Mean Productivity (GMP) [16], Stress Susceptibility Index (SSI) [17], Stress Tolerance Index (STI) [18], and Yield Index (YI) [17] and Yield Stability Index (YSI) [19]. These calculations were performed using Microsoft Excel. The MGIDI index was used to identify drought-resistant genotypes. MGIDI is used in biological experiments for multivariate data analysis, where incorporating multiple traits is crucial for effective genotype selection. However, this process is challenging since traditional linear multi-trait selection indexes exist, but issues such as multicollinearity and the arbitrary assignment of weighting coefficients can undermine genetic gains [5]. Data analysis and selection of the top-performing genotypes for drought tolerance were carried out using the R software package 'metan' [20].

## Limitations

A limitation of this dataset is that it does not completely imitate real-world ground situations. Although the managed situation allows for accurate quantification, the inconsistency in soil types, microclimates, and farming practices present in actual agricultural settings limits the study's applicability. To progress the hands-on relevance of the recognized drought-resilient genotypes, additional validation in various field conditions is essential.

## Ethics Statement

All authors have read and follow the ethical requirements for publication in Data in Brief and our work meets these requirements. Our work does not involve studies with animals and humans.

## CRediT Author Statement

**Nadira Mokarroma:** Conceptualization, Methodology, Investigation, Supervision Writing- Original draft preparation. **Md Romij Uddin:** Investigation, Supervision, Validation. **Imrul Mosaddek Ahmed:** Conceptualization, Methodology, Data curation. **AHM Motiur Rahman Talukder:** Data curation, Software. **Abul Fazal Mohammad Shamim Ahsan, Zakaria Alam:** Writing-Reviewing and Editing.

## Data Availability

Mendeley DataSelection of drought tolerant barley genotypes using stress indices (Original data).

## Appendix Supplementary materials

Image, application 1
