# Supplementary material for: Multivariate analysis for identifying drought-tolerant barley (Hordeum vulgare L.) genotypes using stress indices
Source: Data Brief. 2025 Mar 7;59:111452. doi: 10.1016/j.dib.2025.111452 (PMC11951001; doi:10.1016/j.dib.2025.111452)
Supplement: Supplementary file 1 [file mmc1.docx]

Table S1. Pearson’s coefficients of correlations among studied traits of 50 barley genotypes

| Traits | Yp | Ys | TOL | MP | GMP | SSI | STI | YSI | YI |
| --- | --- | --- | --- | --- | --- | --- | --- | --- | --- |
| Yp | 1.00 |  |  |  |  |  |  |  |  |
| Ys | 0.91*** | 1.00 |  |  |  |  |  |  |  |
| TOL | -0.66*** | -0.92*** | 1.00 |  |  |  |  |  |  |
| MP | 0.96*** | 0.99*** | -0.85*** | 1.00 |  |  |  |  |  |
| GMP | 0.94*** | 1.00*** | -0.88*** | 1.00*** | 1.00 |  |  |  |  |
| SSI | -0.80*** | -0.98*** | 0.97*** | -0.94*** | -0.96*** | 1.00 |  |  |  |
| STI | 0.95*** | 0.99*** | -0.85*** | 0.99*** | 0.99*** | -0.93*** | 1.00 |  |  |
| YSI | 0.80*** | 0.98*** | -0.97*** | 0.94*** | 0.96*** | -1.00*** | 0.93*** | 1.00 |  |
| YI | 0.91*** | 1.00*** | -0.92*** | 0.99*** | 1.00*** | -0.98*** | 0.99*** | 0.98*** | 1.00 |

^Yp^ Yield under control condition, ^Ys^ yield under stress condition, ^TOL^ Tolerance Index, ^MP^ Mean Productivity, ^GMP^ Geometric Mean Productivity mean, ^SSI^ Stress Susceptibility Index, ^STI^ Stress Tolerance Index, ^YSI^ Yield stability index, ^YI^ Yield Index, *** *p* <0.001

Table S2. Description of 50 barley genotypes

| Genotypes | Source | Collected year | Situation | Genotypes | Source | Collected year | Situation |
| --- | --- | --- | --- | --- | --- | --- | --- |
| BD7088 | PGRC, BARI | 2012 | NK | IBON 8 | PBD, BARI | 2005 | NK |
| BD7188 | PGRC, BARI | 2012 | NK | IBON 9 | PBD, BARI | 2005 | NK |
| BD7189 | PGRC, BARI | 2012 | NK | IBON11 | PBD, BARI | 2005 | NK |
| BD7191 | PGRC, BARI | 2012 | NK | IBON12 | PBD, BARI | 2005 | NK |
| BD7192 | PGRC, BARI | 2012 | NK | IBON 13 | PBD, BARI | 2005 | NK |
| BD7193 | PGRC, BARI | 2012 | NK | IBON 14 | PBD, BARI | 2005 | NK |
| BD7194 | PGRC, BARI | 2012 | NK | IBON 16 | PBD, BARI | 2005 | NK |
| BD7195 | PGRC, BARI | 2012 | NK | IBON 19 | PBD, BARI | 2005 | NK |
| BD7196 | PGRC, BARI | 2013 | NK | IBON 21 | PBD, BARI | 2005 | NK |
| BD7197 | PGRC, BARI | 2015 | NK | IBON 24 | PBD, BARI | 2005 | NK |
| BD7202 | PGRC, BARI | 2015 | NK | IBON 26 | PBD, BARI | 2018 | NK |
| BD7203 | PGRC, BARI | 2015 | NK | IBON 28 | PBD, BARI | 2018 | NK |
| BD7204 | PGRC, BARI | 2015 | NK | IBON 36 | PBD, BARI | 2018 | NK |
| BD7205 | PGRC, BARI | 2015 | NK | IBON 37 | PBD, BARI | 2018 | NK |
| BD7206 | PGRC, BARI | 2015 | NK | IBON 52 | PBD, BARI | 2018 | NK |
| BD8572 | PGRC, BARI | 2020 | NK | IBON 54 | PBD, BARI | 2018 | NK |
| BD8573 | PGRC, BARI | 2020 | NK | IBON 59 | PBD, BARI | 2018 | NK |
| BD8574 | PGRC, BARI | 2020 | NK | IBON 68 | PBD, BARI | 2018 | NK |
| BD8579 | PGRC, BARI | 2020 | NK | IBON 86 | PBD, BARI | 2018 | NK |
| BD9194 | PGRC, BARI | 2020 | NK | IBON 97 | PBD, BARI | 2018 | NK |
| BD9680 | PGRC, BARI | 2020 | NK | IBON120 | PBD, BARI | 2018 | NK |
| BD9681 | PGRC, BARI | 2020 | NK | BARI Barley2 | RV | 1994 | HYV, DT |
| BD9682 | PGRC, BARI | 2020 | NK | BARI Barley5 | RV | 2005 | DT |
| BD9683 | PGRC, BARI | 2020 | NK | BARI Barley8 | RV | 2018 | ST |
| BD9684 | PGRC, BARI | 2020 | NK | BARI Barley9 | RV | 2018 | DT |

^NK^ Not known, ^PGRC^ Plant Genetic Resource Center, ^BARI^ Bangladesh Agricultural Research Institute, ^PDB^ Plant Breeding Division, ^RV^ Released variety, ^HYV^ High yielding variety, ^DT^ Drought tolerant, ^ST^ Saline tolerant
